# Modern Radiotherapy for Pediatric Brain Tumors

**DOI:** 10.3390/cancers12061533

**Published:** 2020-06-11

**Authors:** Nicholas J. DeNunzio, Torunn I. Yock

**Affiliations:** Department of Radiation Oncology, Massachusetts General Hospital/Harvard Medical School, 55 Fruit St, Boston, MA 02114, USA; ndenunzio@mgh.harvard.edu

**Keywords:** pediatric brain tumors, childhood, radiation, radiotherapy, treatment planning, proton, IMRT, survival, toxicity, late effects

## Abstract

Cancer is a leading cause of death in children with tumors of the central nervous system, the most commonly encountered solid malignancies in this population. Radiotherapy (RT) is an integral part of managing brain tumors, with excellent long-term survival overall. The tumor histology will dictate the volume of tissue requiring treatment and the dose. However, radiation in developing children can yield functional deficits and/or cosmetic defects and carries a risk of second tumors. In particular, children receiving RT are at risk for neurocognitive effects, neuroendocrine dysfunction, hearing loss, vascular anomalies and events, and psychosocial dysfunction. The risk of these late effects is directly correlated with the volume of tissue irradiated and dose delivered and is inversely correlated with age. To limit the risk of developing these late effects, improved conformity of radiation to the target volume has come from adopting a volumetric planning process. Radiation beam characteristics have also evolved to achieve this end, as exemplified through development of intensity modulated photons and the use of protons. Understanding dose limits of critical at-risk structures for different RT modalities is evolving. In this review, we discuss the physical basis of the most common RT modalities used to treat pediatric brain tumors (intensity modulated radiation therapy and proton therapy), the RT planning process, survival outcomes for several common pediatric malignant brain tumor histologies, RT-associated toxicities, and steps taken to mitigate the risk of acute and late effects from treatment.

## 1. Introduction

Cancer is the second leading cause of death in children aged 1–14 years, with approximately 11,000 diagnoses anticipated in 2020 [1]. Tumors of the central nervous system (CNS) are the most commonly encountered solid tumors in the pediatric population [1]. Most involve the brain, either as a solitary primary lesion or as a primary lesion that disseminates into the cerebrospinal fluid (CSF) and spinal canal. However, depending on the clinical context of disease in each patient (e.g., histology, extent of disease, patient age), treatment paradigms can vary markedly from single-modality therapy to combinations of surgery, systemic therapy, targeted agents, and/or radiation therapy (RT).

The diversity of ways in which RT may be delivered is particularly important to treating patients with curative intent as different techniques have varying ability to minimize dose to normal tissues. It is the dose to normal tissues that causes both acute and late effects of treatment, with the late side effects typically of greater concern as they are usually permanent. Patients are still developing physically and cognitively in combination with having excellent survival and, therefore, have a lifetime to contend with the late side effects of treatment [2].

Radiation exposure to the brain can predispose one to hearing loss, impaired neurocognition, and neuroendocrine dysfunction [3,4,5], among other functional deficits. These toxicities can detract from a child’s quality of life by predisposing survivors to high rates of serious chronic health conditions [6,7,8]. The likelihood of developing toxicity is dependent on the dose of radiation used, younger age at exposure, and volume of tissue irradiated.

To maximize the therapeutic window between efficacy and toxicity, RT has undergone revolutionary changes in recent decades like the development of intensity modulated radiation therapy (IMRT), volumetric modulated arc therapy (VMAT), and proton beam radiation therapy (PBRT). These technologic advances have enabled modification of the type of radiation used as well as the shape and intensity of a given beam. Each allows for improved dose coverage of the tumor and sparing of normal tissues relative to traditional methods such as three-dimensional conformal radiation therapy (3DCRT).

In this review, we summarize the major RT modalities available to treat pediatric brain tumors, describe the modern treatment planning process, and discuss the successes achieved and challenges encountered in treating several of the most common pediatric brain cancers.

## 2. Physical and Dosimetric Principles

The two dominant modalities used in modern pediatric radiation oncology are photon and proton radiation. Though they are produced by different means, a topic that is outside the scope of this review, each is delivered by a beam that originates outside the patient and deposits energy in the patient’s tumor bed and regions at risk for tumor spread. Radiation by either photons or protons causes double-strand breaks in DNA that can impair cell division and results in mitotic catastrophe. To fully understand the strengths and weaknesses of each modality, it is helpful to first consider what happens to each radiation type as it travels along its path from source to tumor.

### 2.1. Depth-Dose Properties: Photons

**Figure 1:**
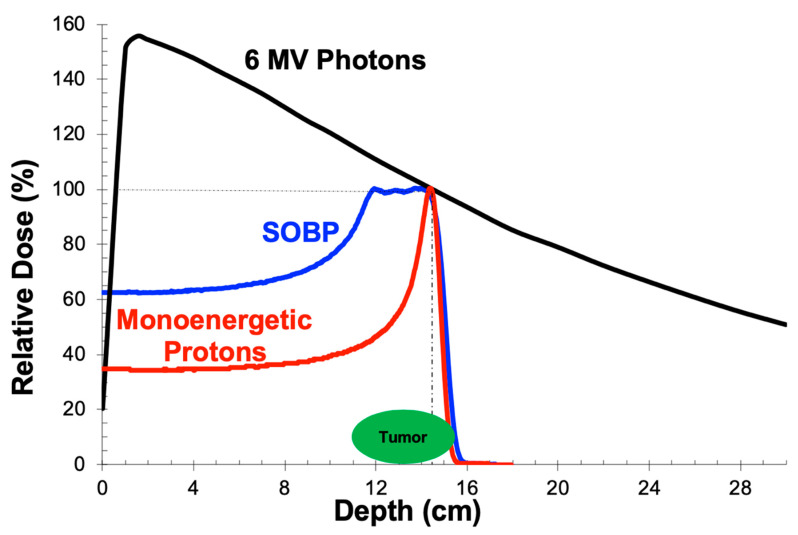
Single beam dose profiles for photons and protons. D_max_, the peak of the 6 MV photon curve (black), occurs at a depth of about 1.5 cm for a 10 × 10 cm field. The characteristic Bragg peak of a monoenergetic proton beam (red) is centered at a depth of 14.5 cm. The distal edge, to the right of the vertical dashed line, is an area of great interest when limiting dose to normal tissues immediately beyond the tumor. The spread-out Bragg peak (SOBP; blue) represents a summation of Bragg peaks from multiple protons with varied energies. This allows tumors (green) to be covered adequately by depositing therapeutic doses of radiation over a longer path.

Photons, more commonly referred to as X-rays, are uncharged entities with wave-particle duality. When produced by a linear accelerator, the machines used for radiation treatment, photons have a spectrum of energies (although the maximum value is referenced). When traveling through tissue, they slowly lose energy and, effectively, deposit dose in all tissues along their path. Dose deposition builds with depth from the skin surface until some distance, d_max_, at which point the maximal tissue dose is achieved relative to the incident beam (Figure 1). D_max_ increases with beam energy such that higher-energy photons can be used to target deeper tumors. Typically, energies between 6 and 15 megavolts (MV) are used for photon RT. Because dose is deposited along the beam path and not just at d_max_, the radiation target (e.g., tumor or tumor bed) as well as surrounding tissues receive significant dose. It is this dose to bystander tissues, including uninvolved organs, that can cause side effects from treatment and an increased risk of a second primary cancer years later [9,10]. Other, more common, late effects are also possible and can have a significant impact on a patient’s quality of life and will be discussed below.

### 2.2. Depth-Dose Properties: Protons

Protons, in contrast to photons, are charged particles with a mass that is nearly 2000 times that of an electron. They have a dose deposition profile that is flat over most of the distance that a proton travels in tissue, but with a hallmark Bragg peak at the very end (Figure 1). This results in lower dose deposition prior to a narrow range in which the bulk of the particle’s energy is deposited. Furthermore, given that all of a proton’s energy is dissipated in the Bragg peak just prior to stopping, there is no dose distal to this Bragg peak (and thus no exit dose is delivered) as would be the case when using photons. As most tumors are larger than the width of a single Bragg peak, the proton beam’s energy is modulated during treatment to generate a spread-out Bragg peak (SOBP, Figure 1). This is a continuum of proton path lengths, each with its own Bragg peak, that are summed to span the depth of a tumor in the same dimension as the beam.

### 2.3. Treatment Delivery: Photons

**Figure 2:**
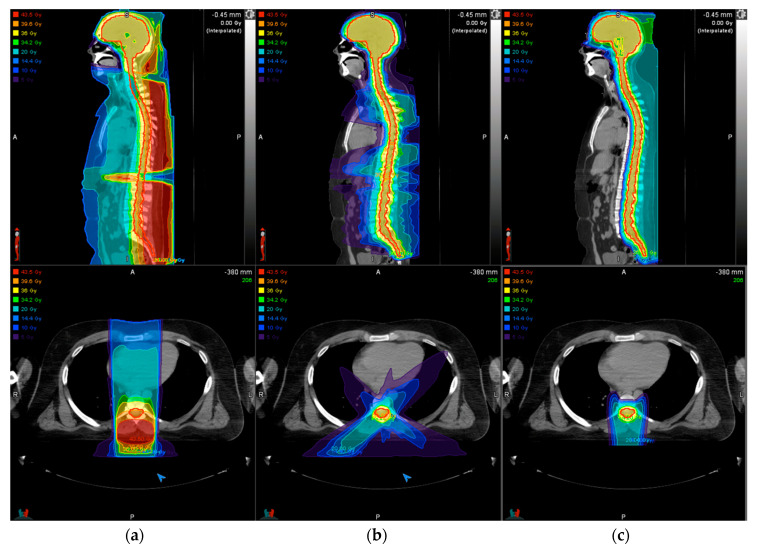
Comparison of dose distributions for a patient undergoing craniospinal irradiation to a dose of 36 Gy by three-dimensional conformal radiation therapy (3DCRT) (**a**), intensity modulated radiation therapy (IMRT) (**b**), or proton beam radiation therapy (PBRT) (**c**). Each pane shows the dose distribution in sagittal (top) and transverse (bottom) views. The target volume is indicated by the thick red line that encompasses the brain and spinal canal down to the thecal sac. Prescription dose (shaded yellow) and target coverage are similar across modalities, but higher entrance (posterior) dose is seen in the 3DCRT plan in comparison with IMRT or PBRT. The abdomen receives more low-dose radiation (shaded green, blue, and purple) using photon modalities compared with PBRT.

Linear accelerators deliver treatment through a variety of means, each with its strengths and weaknesses. Simpler treatments are forward planned, meaning that beams are manually selected and positioned by the radiation dosimetrist or physicist, to cover the target tissue and calculate the dose received by both the radiation target and nearby tissues. This can be reiterated with the radiation oncologist until achieving the desired balance of dose to the tumor and surrounding tissues. Treatment plans can be generated relatively quickly, generally in one to two days. However, these plans are not as conformal as others discussed below (Figure 2). This technique is less optimally used when the target is in close proximity to healthy organs that cannot tolerate much radiation dose or when the target has an irregular geometry such that optimal coverage of concavities or convexities results in higher doses to surrounding tissues.

Inverse planning through IMRT or VMAT, however, allows for coverage of irregularly shaped targets by specifying the radiation prescription dose and coverage up front as well as dose limits for normal tissues. These are collectively referred to as treatment objectives. IMRT is achieved through modulating the intensity of the radiation beam during treatment delivery. In contrast to fixed-field or “step and shoot” IMRT, whereby radiation is delivered at (five to nine) fixed beam positions, VMAT delivers IMRT by modulating the intensity of the radiation beam continuously during treatment while the machine rotates around the patient and thereby shortens the treatment time. Treatment objectives are turned over to a computerized optimization algorithm that generates a plan that optimizes the radiation dose distribution relative to the treatment objectives. The advantage of these approaches to photon RT compared with 3DCRT is exquisite customization of the radiation dose distribution given any patient’s anatomy using equipment that is widely available. The disadvantages, however, are that treatment planning takes significantly longer (anywhere from one to two weeks) and that a low dose of radiation to a greater volume of normal tissues occurs (Figure 2).

### 2.4. Treatment Delivery: Protons

Protons used in radiation oncology are generated by a cyclotron or synchrotron, which, until recently, were too expensive for the technology to be widely adopted. However, technical advances have allowed production of more compact units that fit within a single treatment room and have much lower start-up costs. Currently, there are over 30 operating proton beam facilities in the United States, with more planned in the coming years [11].

There are two main modalities by which protons are delivered: passive scatter (PS) and pencil beam scanning (PBS). These are analogous to delivery of photons via 3DCRT and IMRT, respectively. However, these proton delivery techniques have different hardware requirements. Though there remain circumstances when PS may be preferred, PBS generally provides better sculpting of the delivered dose because of the ability to shape the dose deposition both to the distal edge of the tumor as well as the proximal edge. PS techniques can only shape the beam to the distal edge. Conformality with PS comes from using multiple beams (usually three or four for a brain tumor). PS techniques require brass apertures and tissue compensators to be crafted for every beam that is used. The pencil beam techniques require neither, although it can benefit from brass apertures to improve lateral penumbra (dose fall-off). Furthermore, PBS technology is a prerequisite to using intensity modulation of the proton beam, which cannot be done with PS techniques. Given the advantages of pencil beam technology, the newest treatment centers deliver protons exclusively by PBS. The dose distributions achieved using either PS or PBS proton radiation are superior to those seen with photon delivery techniques, with the benefit of less entrance and no exit dose compared with photon beams (Figure 2). This results in less integral dose, the dose received by the entire body and not just the target volume, and thereby diminishes the extent of acute and late effects from treatment and has the potential to diminish the risk of second neoplasms based on modeling studies [12,13,14,15,16]. Additional follow up/clinical data are needed to confirm these findings.

## 3. Treatment Planning

Despite the physical and dosimetric properties of the treatment modalities used in modern RT, treatment of brain tumors (as well as many other malignancies) follows a similar work flow that includes a computed tomography scan obtained in the treatment position (TPCT), target delineation, and radiation plan generation.

Given the highly conformal nature of the techniques used to treat pediatric brain tumors, acquisition of diagnostic-quality three-dimensional imaging is imperative as it is these images that largely define the precision with which radiation targets and normal tissues can be delineated. Magnetic resonance imaging (MRI) is the best imaging modality for brain tumors in both the pediatric and adult populations. Typically, both the diagnostic scans and a treatment planning MRI are used to optimally plan the RT for a pediatric brain tumor patient. These MRIs are obtained in their digital imaging communications in medicine (DICOM) format to co-register with the radiation TPCT scan. It is thus helpful if these images are obtained in a volumetric manner with thin slices (≤3 mm) and no skips.

Because it is important that the patient be immobilized for both image acquisition and treatment, children may require anesthesia for both the TPCT and daily treatments. Typically, children six years of age and older can lie still for 20 min while a treatment is delivered. However, every child is different and some, owing to anxiety or neurologic complications of surgery, require daily anesthesia for treatment at older ages or if the treatment is prolonged. Behavioral interventions and the help of a child life specialist can help younger patients follow instructions, obviate the need for anesthesia, and reduce health care costs [17,18]. Avoiding daily anesthesia means allowing the patient access to nutrition for greater time during the day, which has clear health benefits.

**Figure 3:**
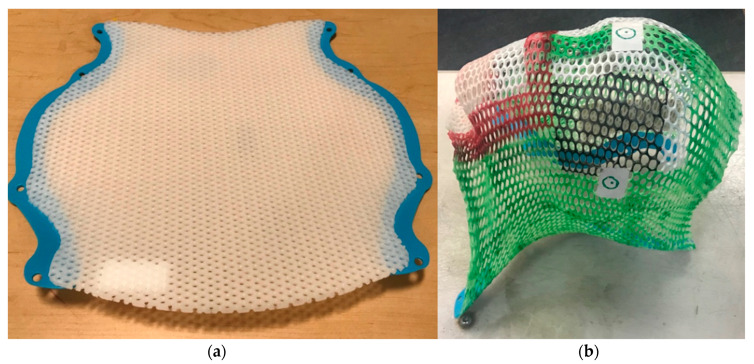
Immobilization device for RT. To create an immobilization device for each patient undergoing RT to treat a brain tumor, a hard sheet of plastic mesh (**a**) is warmed in a water bath before stretching it to conform to the contours of the head. The mesh then cools and hardens, forming a customized mask (**b**) that can be fixed to the table on which the patient lies during daily radiation treatments.

The patient is then carefully positioned using orthogonal lasers mounted on the room walls. Next, a thermoplastic mask is shaped by stretching a warm and wet sheet of plastic mesh over the head such that, when it cools, it conforms to facial features and can be affixed to the head positioner to immobilize the head (Figure 3). Marks are then placed on the mask that serve as landmarks for daily treatment setup in the treatment room, where lasers are used as well. Finally, the TPCT is acquired with thin slices (≤2.5 mm thick) with or without intravenous contrast. Setup images and measurements are recorded to aid the accuracy of daily treatment setups.

TPCT images are then registered with the diagnostic brain imaging acquired as noted above. The target(s) and normal anatomic structures are delineated. Of note, aligning and co-registering images can be more complicated in pediatric patients depending on a patient’s growth rate, time interval between pretreatment scans and radiation planning, and if scans are obtained in different positions.

The radiation oncologist then works with the physics and/or dosimetry staff to draft a treatment plan that optimizes the balance between providing an appropriate dose to the target(s) while minimizing doses to surrounding organs and tissues. The physician reviews the draft plan by examining the three-dimensional dose coverage over the target volumes and by inspecting a dose volume histogram (DVH) that plots a curve of volume versus dose received for each target or organ that has been contoured. At this point, the radiation oncologist considers a patient’s entire clinical picture, DVH metrics, goals for treatment, and potential for late effects in deciding whether adjustments need to be made. These last two steps of treatment planning between physics and physicians comprise an iterative process. The approved radiation plan then undergoes a series of tests for quality assurance before the patient starts treatment. In total, radiation planning takes one to two weeks to complete, depending on what type of planning is employed.

## 4. Treatment Outcomes for Select Histologies

Long-term survival after treatment of pediatric brain tumors is excellent at approximately 75% [2]. Though pediatric brain tumors encompass a wide spectrum of histologies, those featured here are those most frequently encountered and commonly require radiotherapy as part of definitive management. Surgery and/or chemotherapy may also play a role depending on histology, patient age, and tumor location.

### 4.1. Medulloblastoma

**Figure 4:**
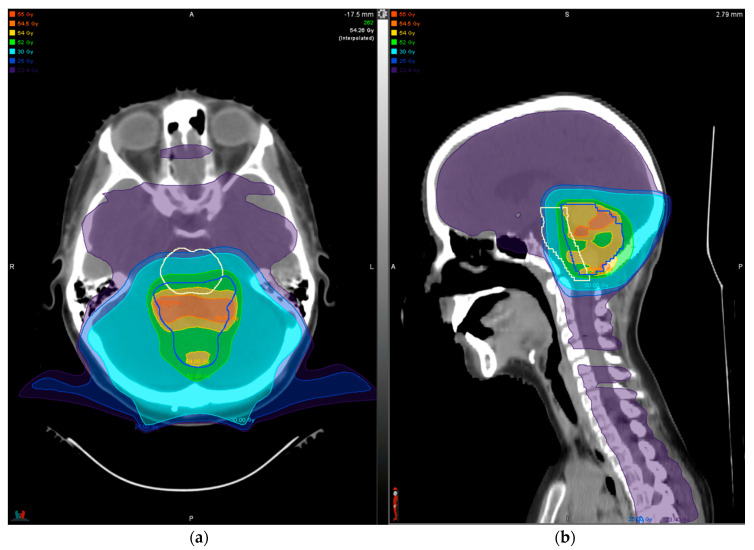
Dose distribution for treatment of standard-risk medulloblastoma shown in axial (**a**) and sagittal (**b**) views. A dose of 23.4 Gy (shaded purple) was delivered to the entire craniospinal axis, followed by 30.6 Gy to the tumor bed only (darkest blue line) for a total dose of 54 Gy (shaded yellow) to the tumor bed. Delivery of the prescription dose to the entire tumor bed is limited owing to proximity of the brainstem (white line), such that the entire target receives nearly the full 54 Gy. Note that multiple posterior beams from varied angles are used (**a**) to distribute dose deposited at the end of range near the brainstem.

Medulloblastoma is the most common embryonal CNS tumor in the pediatric population. It presents as a mass in the posterior fossa that often causes hydrocephalus from impeding CSF circulation. Treatment in children three years of age and older consists of surgery followed by RT, which includes both craniospinal irradiation (CSI) with a higher dose to the tumor bed. The CSI prescription dose depends on whether the patient has standard-risk (23.4 Gray [Gy]) or high-risk (36 Gy) disease, though the total dose delivered to the tumor bed is the same (54 Gy) with treatment delivered over six weeks, or 30 sessions (Figure 4). Multi-agent chemotherapy is also given in the adjuvant setting, although most patients also receive weekly vincristine during RT. Molecular classification of medulloblastomas shows that the disease falls into one of four groups: wingless (WNT), sonic hedgehog (SHH), group 3, and group 4. These groupings have prognostic implications, with WNT patients having the best prognosis and group 3 having the worst [19].

### 4.2. Ependymoma

Ependymomas are common pediatric brain tumors in young children, with a median age of four years at the time of presentation. Approximately two-thirds arise in the posterior fossa and one-third arise in the supratentorial space. The two histologies that occur in the brain are World Health Organization (WHO) grade II (classic) and grade III (anaplastic) ependymomas, with the latter typically associated with a worse prognosis. The mainstay of treatment in children with localized disease is complete surgical resection followed by RT (54–59.4 Gy) to the tumor bed. Between 5% and 10% of patients present with dissemination within the CNS and, if three years of age or older, require CSI as part of their treatment regimen. Group A (EPN_PFA) and 1q gain are poor prognostic factors in the posterior fossa tumors, while the RELA molecular subtype confers a worse prognosis in the supratentorial tumors [20,21,22]. Children’s Oncology Group (COG) evaluated outcomes of patients treated with post-operative RT for pediatric ependymomas in study ACNS0121. This demonstrated a five-year event free survival rate of 65–70% after gross or near-total resection of the tumor compared with 37% for tumors that were incompletely resected with adjuvant chemotherapy [21]. The role of adjuvant chemotherapy is under active investigation in ACNS0831, the most recent ependymoma protocol from COG.

### 4.3. Glioma

Pediatric gliomas arise from glial cells in the CNS and are varied in their histology and anatomic distribution. Definitive management of low-grade gliomas (LGG) ideally involves gross total resection with local control rates of about 80% and survival rates ≥95% at five years from treatment [23,24,25]. However, many LGGs cannot be resected owing to the involvement of critical structures such as the hypothalamus, optic pathway, or brainstem. For unresectable tumors, RT can be used alone or after chemotherapy as a bridge to RT. Because patients often respond to chemotherapy, typically, children under age ten are treated with chemotherapy prior to RT. Modern RT techniques like IMRT and PBRT seek to minimize the side effects associated with radiation exposure to eloquent parts of the brain. However, it is important to highlight that a clinical benefit from using PBRT with respect to a given organ is not possible to achieve when that organ is infiltrated with tumor. An example of this is when attempting to spare the hypothalamus when treating a hypothalamic glioma. Proton techniques are helpful in sparing normal tissues nearby, but cannot spare structures involved with tumor [26]. In this example, protons and photons will give an equal dose to the hypothalamus, but the nearby hippocampi should be better spared with proton techniques.

## 5. Late Effects

Given high long-term survival rates, understanding and documenting late effects stemming from treatment of pediatric brain tumors is important to improving outcomes. It is a concomitant focus on improving survival while optimizing quality of life that has spawned several ongoing efforts within the pediatric radiation oncology community. These include collaborations across institutions such as through the Pediatric Proton/Photon Consortium Registry (PPCR) [27,28] and Pediatric Normal Tissue Effects in the Clinic (PENTEC) [29], among others. Late effects stemming from RT for pediatric brain tumor patients are dependent on anatomic location of the radiation target and can include neurocognitive dysfunction, neuroendocrine dysfunction, ototoxicity, brainstem toxicity, alopecia, and second malignant and non-malignant neoplasms [3,5,9,30,31,32,33,34]. Each of these will be considered in turn.

### 5.1. Neurocognition

PBRT is often advocated for in young children with brain tumors because of its ability to spare brain tissue and mitigate the adverse effects of RT on the developing brain, including impaired neurocognition. However, PBRT is expected to improve neurocognitive outcomes compared with photon treated patients based on clinical modeling [26].

This is supported by retrospective studies of radiation dosimetry of the hippocampus/temporal lobe and cerebellum and clinical outcomes [35,36,37]. In addition, data from a phase II study of pediatric patients with medulloblastoma treated with PBRT at Massachusetts General Hospital (MGH) show a decline in full-scale intelligence quotient (FSIQ) of 1.5 points per year over a median follow up of just over five years [34], which is less than the annual decrements reported in photon-treated cohorts that range from 1.9 to 5.8 points [38,39].

A more direct comparison of photon and proton treatment modalities was made in a retrospective study of patients with medulloblastoma treated on prospective protocols at Texas Children’s Hospital (PBRT delivered at MD Anderson) and The Hospital for Sick Children (Toronto; photon RT delivered as PBRT was not available in Canada) [40]. PBRT was associated with better global IQ scores and scores in the perceptual reasoning and working memory domains over long-term follow up. In addition, for patients treated with PBRT, all tested IQ domains were stable over time, except for processing speed.

### 5.2. Neuroendocrine Function

Effects on neuroendocrine function are the result of the radiation dose received by both the hypothalamus and pituitary and are relevant to treatment of a wide variety of tumors, including (optic pathway) gliomas, medulloblastomas, and ependymomas. In a mixed brain tumor cohort of patients treated with protons at MGH, a combined dose to the hypothalamus and pituitary ≥40 Gy places patients at significant risk of developing hormone deficiency in the years following therapy, most commonly growth hormone [5]. Other risk factors include age under ten years at the time of treatment and time since therapy. Furthermore, there is variable latency and cumulative incidence of developing deficiency among each of the pituitary hormones. Similar findings were reported by St. Jude from treating low grade gliomas with photons predominantly in the diencephalon [3]. Growth hormone and thyroid hormone levels have consistently been reported to be the most radiosensitive, but cortisol and the sex hormones can also be affected. Therefore, screening is warranted for all four hormone axes for several years after completing RT.

### 5.3. Hearing

Ototoxicity from RT is possible depending on tumor proximity to the cochleae and is particularly relevant to the treatment of tumors of the posterior fossa (infratentorial ependymomas and medulloblastomas). A study of localized brain tumors treated with RT (and without exposure to chemotherapy) at St. Jude demonstrates that a mean cochlear dose <30 Gy is associated with a low risk of cochlear dysfunction, while doses >40 Gy increase the risk of hearing loss, especially at high frequencies [41].

Studies of RT-associated ototoxicity in the treatment of medulloblastoma have been conducted. The rates of ototoxicity after photon RT range from 18% to 24% [4,42], while a study from MGH using PBRT demonstrated a lower rate (16% for grades 3 and 4 on the Pediatric Oncology Group scale) five years after treatment [34].

In contrast, a retrospective study of children treated for medulloblastoma at Texas Children’s Hospital and MD Anderson showed no difference in clinical outcome between photon RT and proton modalities despite lower mean cochlear dose achieved when using protons [43]. Patients received CSI, sequential RT boost to the posterior fossa and/or tumor bed (photons) or the tumor bed only (protons), and adjuvant cisplatin-based chemotherapy. The ototoxicity from cisplatin, however, may be responsible for the similar rates of ototoxicity observed considering the aforementioned dose constraints. The mean cochlear doses in this study were 37.3 Gy for the photon group and 31.5 Gy for the proton group.

### 5.4. Cerebrovascular Effects

#### 5.4.1. Moyamoya

Pediatric patients who receive cranial RT can develop vasculopathies of small and large arteries years to decades after completing treatment. The most common large-vessel vasculopathy is moyamoya. This is non-atherosclerotic stenosis of the intracranial carotid arteries and their proximal branches, leading to reduced perfusion and formation of collateral vessels with aberrant architecture. This pathology develops in 3–4% of patients receiving cranial RT, with an onset as early as one year after treatment [44,45]. Clinical presentations are varied and include, but are not limited to, transient ischemic attack/stroke, cognitive impairment, headache, seizure, and choreiform movements (when in the basal ganglia).

Risk factors are both environmental and genetic in nature, including age (moyamoya has a bimodal distribution overall with peaks at five and ~45 years of age), tumor abutment of and radiation dose delivered to the circle of Willis (CW), neurofibromatosis type 1, and Down’s syndrome [44]. For patients who receive an RT dose ≥50 Gy (a dose range commonly used when treating primary brain tumors) to the optic chiasm, and thus the CW by proximity, 4–10% will develop moyamoya after eight years from treatment [45].

#### 5.4.2. Cavernomas and Cerebrovascular Bleeds

Cavernomas and cerebral microbleeds (CMBs) are thin-walled clusters of dilated capillaries or venules that can occur spontaneously, but are found more commonly in children who have a history of cranial radiation. They can range in size from less than one millimeter to greater than one centimeter and are most often asymptomatic and of little clinical consequence. However, the larger cavernomas can occasionally bleed and cause headache, focal neurologic deficit/stroke, seizure, or impaired cognition, depending on their location [46,47]. For CMBs associated with prior RT, their incidence directly correlates with treatment volume such that a higher rate of CMBs is seen in patients with a history of whole brain RT compared with focal irradiation. Multiple studies have demonstrated an increasing prevalence of CMBs with time since RT, often within the first few years after treatment as well as RT dose received, history of chemotherapy, and use of antithrombotic therapy [47,48].

The true cumulative incidence of RT-induced cavernomas is not precisely known, as their detection depends on their size and the MRI technique used. CMBs are typically very small and low-contrast relative to normal brain parenchyma. Iron-sensitive MRI sequences have been developed to improve their detection. These include T2 gradient echo (T2-GRE) and susceptibility weighted imaging (SWI), which have been found to augment detection by 50–70% by enhancing contrast between brain parenchyma and paramagnetic materials such as deoxyhemoglobin, hemosiderin, and calcium [49]. Estimates vary greatly, from 14% at 15 years after treatment using standard/non-iron sensitive T1 and T2 MRI sequences [50] to >60% at a median of four years after treatment using T2-GRE sequences [48].

#### 5.4.3. Stroke

Data are emerging that demonstrate a risk of late stroke in survivors of pediatric brain tumors treated with radiation. The cumulative incidence of clinical stroke after cranial radiation is 2% at five years and 4% at ten years after treatment [51]. Stroke risk is dependent on tumor histology, radiation dose, and time since RT. The cumulative incidence approaches 15% in 30-year survivors who have received >50 Gy [51,52,53,54]. Risk of stroke after RT to the CW also increases with elapsed time and radiation dose [53]. Other risk factors for a first stroke include hypertension, diabetes mellitus, and black race. Risk of subsequent stroke is significantly higher in patients who have suffered an initial stroke with estimates ranging from 25% to 59% at ten years after the initial stroke and a median time to second stroke of 15 months [51,55]. Intensified risk reduction strategies may be warranted in patients with multiple risk factors.

#### 5.4.4. Post-Treatment Surveillance

Given the short- and long-term risks of radiation-induced vasculopathies in survivors of childhood cancer who received cranial radiotherapy, these authors advocate baseline MR angiogram (MRA) and SWI, which can be performed in conjunction with the radiation planning MRI. The first post-treatment surveillance MRI is also a reasonable time to obtain these baseline studies as development of treatment-associated vasculopathy or cavernomas usually occurs over months to years. Occasionally, owing to tumor compression or surgical intervention, early imaging reveals tumor-related vasculopathies that should be monitored. MRA and SWI should be obtained annually or semi-annually with regular surveillance MRIs in patients receiving ≥50 Gy to a portion of the CW.

### 5.5. Relative Biologic Effectiveness and Brainstem Toxicity

Because many pediatric brain tumors, including medulloblastomas and infratentorial ependymomas, arise in proximity to the brainstem and its exiting cranial nerves, surgical resection can be both challenging and/or morbid. Gross residual disease near the brainstem presents challenges for the radiation oncologist who will want to increase the dose to residual disease, but must also be cognizant of the risk of RT-induced brainstem toxicity. Based on Quantitative Analyses of Normal Tissue Effects in the Clinic (QUANTEC), there is a low risk of toxicity if mean doses are kept to 54 Gy or less. They note that only a very small portion of the brainstem can be brought to a maximal dose of 59 Gy [56]. However, there is controversy around this issue of brainstem tolerance to radiation and whether this differs between proton and photon modalities. It is becoming more widely accepted that the brainstem radiation dose constraints need to be more conservative when using protons compared with photons [30,31]. The current COG ependymoma protocol, ANCS0831, has different dose constraints for the two modalities with a goal of 50% of the brainstem receiving no more than a dose of 61 Gy when using photons or 52.4 Gy when using protons. Though this remains an area of active debate, it is the opinion of these authors that it is more prudent to apply the more conservative proton dose constraints in patients treated with photons as well, but of utmost importance when treating with protons [31,57,58,59].

One hypothesis for why patients treated with PBRT may be more susceptible to brainstem toxicity is that the relative biological effectiveness (RBE) of a proton varies over the course of its path, with most energy and dose being deposited at the distal end of the Bragg peak (Figure 1). Consequently, it is here at the “end of range” where protons have the greatest biologic impact. This poses a challenge in targeting tissues of the posterior fossa where tumor often abuts the brainstem, placing the interface in this critical region where the biologic effect is more pronounced. Therefore, a dose with greater biologic effect may be deposited in the brainstem than is accounted for by photon treatment planning algorithms. To mitigate this risk, multiple beam angles can be used to expand the surface over which dose is deposited at the end of range (Figure 4). Another possibility is to incorporate a model of the biologic dose into the treatment planning process, an approach that has been recently adopted by the Mayo Clinic [60]. Importantly, however, additional factors can contribute to the risk of RT-induced brainstem toxicity, including surgery, chemotherapy, and patient genetics.

### 5.6. Alopecia

The potential for permanent radiation-induced alopecia (RIA) is important in peripherally located tumors and when radiation doses of 50–60 Gy are needed. This happens more commonly in the pediatric glioma population. The dose tolerance of hair follicles varies by patient and chemotherapy exposure, but is approximately 40 Gy. Similar scalp dose constraints to limit the risk of permanent RIA have been reported for photon and proton treatment modalities [32,33]. Importantly, RIA has been associated with increased anxiety in childhood cancer survivors, while head and neck disfigurement has been associated with increased depression [61]. Both photon and proton modalities can be manipulated to spare dose to the skin follicles.

### 5.7. Second Neoplasms

Although the greatest attention is paid to monitoring for recurrent or progressive disease in the first two decades after treatment of any pediatric cancer, a significant cause of late mortality after this time is development of a second malignant neoplasm (SMN) [62]. The rate of SMN development has been estimated by the Childhood Cancer Survivor Study (CCSS) to be 3% in all survivors of pediatric cancers after 20 years of follow up and almost 8% after 30 years [63,64]. As RT is a focal treatment, radiation-associated second tumors will arise within the field of irradiated tissues. The CCSS has shown that the risk of second neoplasms in brain tumor survivors is related to the dose of radiotherapy received, with meningioma histology more likely than high-grade glioma [9]. Volume of tissue irradiated has also been correlated with risk of a second neoplasm, as discussed above in comparing the physical characteristics of photons and protons.

## 6. Conclusions

Treating pediatric brain tumors with RT is a technically complex endeavor with respect to treatment planning and delivery. The process has evolved dramatically in recent decades to enable better definition of radiation targets by effectively leveraging high-quality diagnostic imaging and improved delivery of increasingly conformal treatment through computer-assisted planning and particle/proton therapy modalities. This improved conformity contributes to lower rates of acute and late effects from RT, which is tied to improvements in quality of life in this population where long-term disease survival can be readily achieved in the background of actively developing neural tissues. Furthermore, collaborative efforts established through the PPCR and PENTEC initiatives will help guide further refinement of existing pediatric cancer treatment paradigms to enable pediatric brain tumor survivors to live longer, healthier lives.
